# Influence of Uterine Balloon Tamponade Prevalence on Uterine Artery Embolization in the Management of Postpartum Hemorrhage

**DOI:** 10.3390/jcm15020416

**Published:** 2026-01-06

**Authors:** Hitomi Imafuku, Kenji Tanimura, Sonoko Suda, Naohisa Masuko, Akiko Uchida, Masashi Deguchi, Koji Sasaki, Masato Yamaguchi, Yoshito Terai

**Affiliations:** 1 Department of Obstetrics and Gynecology, Kobe University Graduate School of Medicine, Kobe 650-0017, Japan; ima1210@med.kobe-u.ac.jp (H.I.); sonochame012009@yahoo.co.jp (S.S.); naohisa0115@gmail.com (N.M.); akiko1007libra@yahoo.co.jp (A.U.); deguchi@med.kobe-u.ac.jp (M.D.); yterai@med.kobe-u.ac.jp (Y.T.); 2 Department of Radiology, Kobe University Graduate School of Medicine, Kobe 650-0017, Japan; ssk2ds@gmail.com (K.S.); masato03310402@yahoo.co.jp (M.Y.)

**Keywords:** postpartum hemorrhage, treatment success rate, uterine artery embolization, uterine balloon tamponade

## Abstract

**Objectives:** The aim of our retrospective cohort study was to assess the influence of uterine balloon tamponade (UBT) prevalence on uterine artery embolization (UAE) in management of postpartum hemorrhage (PPH). **Methods:** This retrospective cohort study analyzed, as the final cohort, women with PPH who were transferred from other hospitals or clinics and underwent UAE at our university hospital. Initial UAE success was defined as achieving hemostasis with the first UAE procedure. The use rates of UAE, UAE success rates, and UAE procedural times were compared between the pre-UBT period (January 2009–December 2014) and the UBT period (January 2015–December 2023). **Results:** In the pre-UBT period, 29 of 41 women with PPH underwent UAE. In the UBT period, 21 of 121 women received UAE following UBT, whereas 11 underwent UAE without prior UBT. The use rate of UAE was significantly higher in the pre-UBT period than in the UBT period (70.7% vs. 26.4%; *p* < 0.01). There were no significant differences in the initial (72.4% vs. 81.3%; *p* = 0.55) or final (82.8% vs. 87.5%; *p* = 0.72) success rates of UAE between the two periods. The procedural time of the initial UAE in the UBT period tended to be longer than that in the pre-UBT period (46 min vs. 61 min; *p* = 0.07). **Conclusions:** The introduction of UBT was associated with a reduced use rate of UAE, but it did not significantly affect the success rate or procedural time of UAE in the management of PPH.

## 1. Introduction

Postpartum hemorrhage is a leading cause of maternal morbidity and mortality, accounting for approximately 8% and 20% of maternal deaths in developing and developed countries, respectively [1,2]. According to the American College of Obstetricians and Gynecologists, postpartum hemorrhage is defined as a cumulative blood loss of at least 1000 mL or blood loss associated with signs or symptoms of hypovolemia within 24 h after childbirth [3]. In clinical practice, blood loss of >500 mL after vaginal delivery should be considered abnormal and may require intervention [4,5]. The underlying causes of postpartum hemorrhage are categorized using the “4Ts”: tone (uterine atony), trauma (lacerations or uterine rupture), tissue (retained placenta or clots), and thrombin (clotting factor deficiency) [2,6,7,8]. Uterine atony is the most common cause of postpartum hemorrhage, accounting for approximately 70% of cases, followed by obstetrical lacerations (approximately 20%), retained placental tissue (approximately 10%), and clotting factor deficiencies (<1%) [2,6,8].

The primary approach to managing postpartum hemorrhage focuses on addressing the underlying causes, including removing blood clots, performing bimanual uterine massage, administering uterotonic medications for “Tone” cases [6,7,9,10,11], performing surgical repair for “Trauma” cases [2,7,9], removing retained placenta for “Tissue” cases [7,9,11], and transfusing fresh frozen plasma for “Thrombin” cases [7,12]. If these first-line treatments fail, second-line invasive interventions, including uterine artery embolization, uterine compression sutures, and hysterectomy, are considered [7,11,12]. Uterine artery embolization has become a standard treatment for postpartum hemorrhage, because it is minimally invasive compared with other second-line procedures, has a high success rate, and preserves the uterus [7,8,13,14]. However, uterine artery embolization causes uterine ischemia, infarction, and even necrosis [8,13,15]. Furthermore, it may increase the risk of placenta accreta spectrum in subsequent pregnancies [16].

Particularly in postpartum hemorrhage cases due to “Tone”, when bimanual uterine massage and uterotonic medications fail to control bleeding, uterine tamponade may be considered. In 2012, the World Health Organization recommended uterine balloon tamponade for the management of postpartum hemorrhage prior to initiating invasive procedures [12]. If an intrauterine balloon is not readily available, multiple large Foley catheters may also be used. In Japan, the Bakri^®^ Postpartum Balloon (COOK MEDICAL, Bloomington, IN, USA) was approved for insurance coverage in April 2013, and uterine balloon tamponade was first documented in the Japanese guidelines for managing severe obstetrical hemorrhage in 2017 [17]. Subsequently, in April 2020, the Atom uterine hemostatic balloon (Atom Medical Corporation, Tokyo, Japan) was launched in Japan. Additionally, the Jada^®^ System (Organon, Jersey City, NJ, USA), one of the newest technologies, was approved by the Food and Drug Administration in 2020.

Prior to the introduction of uterine balloon tamponade, uterine artery embolization was performed in cases where hemostasis could not be achieved through first-line treatments, including bimanual uterine massage and administration of uterotonic agents. Conversely, following the introduction of uterine balloon tamponade, uterine balloon tamponade is used in cases where bleeding cannot be controlled by first-line treatments, whereas uterine artery embolization is considered only when hemostasis cannot be achieved with uterine balloon tamponade.

This study aimed to investigate how the widespread adoption of uterine balloon tamponade has influenced uterine artery embolization in the management of postpartum hemorrhage by comparing outcomes before and after its introduction, including the rates of use, success rates, and procedural times.

## 2. Materials and Methods

### 2.1. Study Design and Participants

The present study was conducted in accordance with the principles stipulated in the Declaration of Helsinki and was approved by the Institutional Review Board of Kobe University Graduate School of Medicine (Reference No. B240133).

Between January 2009 and December 2023, women who were transferred from other hospitals or clinics due to postpartum hemorrhage (PPH) to our university hospital were enrolled in the present retrospective cohort study. PPH was defined as a cumulative blood loss of at least 1000 mL or blood loss associated with signs or symptoms of hypovolemia within 24 h after childbirth [3]. Exclusion criteria were as follows: (1) women who initially underwent hysterectomy; (2) women with PPH caused by ‘Trauma’, for which UBT is ineffective; and (3) women who died before receiving hemostasis.

**Figure 1:**
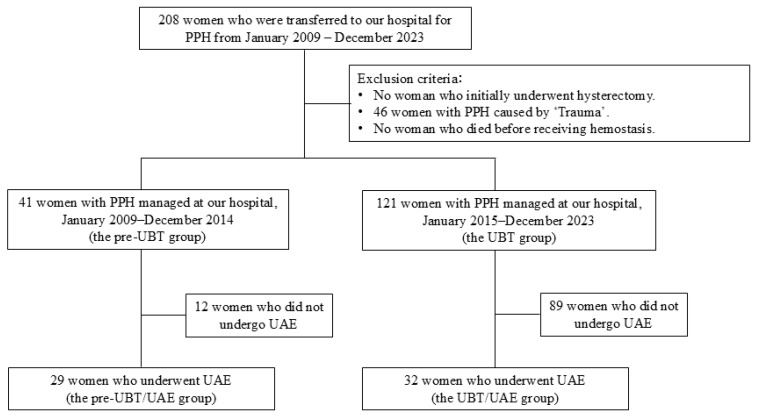
Flowchart of participant selection. Abbreviations: PPH, postpartum hemorrhage; UBT, uterine balloon tamponade; UAE, uterine artery embolization.

In our institution, uterine balloon tamponade (UBT) was introduced in January 2015, and, since then, we have strongly recommended its use to referring hospitals and clinics when transferring patients with PPH. The referring facilities were all within a one-hour ambulance transfer time, with most transfers taking 10–30 min. Accordingly, the study period was divided into the following two phases: (1) pre-UBT period (January 2009–December 2014) and (2) UBT period (January 2015–December 2023). Women who underwent uterine artery embolization (UAE) during the pre-UBT period were categorized as the pre-UBT/UAE group, and those who underwent UAE during the UBT period were classified as the UBT/UAE group. The flow chart illustrating the participant selection process for the study cohort is presented in Figure 1.

The pre-UBT/UAE group served as the control cohort and was compared with the UBT/UAE group to evaluate the effect of introducing UBT on the UAE success rate and procedure time.

### 2.2. Procedures

Upon transfer to our hospital, a team of 2 to 5 obstetricians, including at least one with more than 8 years of experience, assessed the severity of bleeding, determined the cause of PPH, and administered first-line treatments as previously described. Blood transfusions were performed as needed to manage hypovolemic shock or disseminated intravascular coagulation.

During the pre-UBT period, patients with persistent PPH on hospital arrival underwent UAE if their vital signs could be stabilized through blood transfusion. Those presenting with life-threating massive hemorrhage and persistent hemodynamic instability despite blood transfusion underwent hysterectomy.

During the UBT period, in PPH cases where UBT had been placed at the referring facility and hemostasis was confirmed upon arrival at our hospital, UBT was considered effective and no further interventions were performed. In cases where UBT had not been placed and non-massive bleeding persisted upon arrival, we inserted UBT at our hospital. However, in cases with persistent massive hemorrhage, UBT was bypassed. UAE was directly performed in patients whose vital signs were stabilized with blood transfusion or hysterectomy was conducted in those with life-threatening shock despite receiving transfusions.

In cases of UBT-resistant PPH, UAE was performed by experienced radiologists under local anesthesia. A 4- or 5-French sheath was inserted via the left or right common femoral artery. The bilateral uterine arteries were selectively catheterized and embolized with gelatin sponge particles (Serescue, Nippon Kayaku, Tokyo, Japan), which were cut into 1.0–2.0-mm cubes using a scalpel and scissors. In some cases, a mixture of n-butyl-2-cyanoacrylate (Histoacryl, B. Braun, Melsungen, Germany) and iodized oil (Lipiodol, Guerbet, Aulnay-sous-Bois, France) was used. To verify hemostasis, digital subtraction angiography and gynecological examinations immediately were performed post-procedure. If necessary, additional embolization of the ovarian or round ligament arteries was conducted.

If uterine bleeding recurred after the initial UAE procedure, a repeat UAE was performed in patients whose vital signs could be stabilized with blood transfusion. However, hysterectomy was performed in patients with life-threatening recurrent hemorrhage who remained hemodynamically unstable despite receiving transfusions, or when repeat UAE failed to achieve hemostasis.

The patients’ characteristics and clinical data, including age, parity, history of assisted reproductive technology (ART), gestational weeks (GWs), mode of delivery, PPH treatments received at previous hospitals or clinics, estimated blood loss upon arrival at our hospital, and total estimated total blood loss until PPH was controlled, were collected from their medical records. Additionally, the maternal hemoglobin levels and plasma fibrinogen concentrations at hospital arrival, their lowest values before hemostasis, volume of transfused red blood cells (RBCs) and fresh frozen plasma (FFP), and UAE duration were recorded. The UAE procedural time was defined as the interval from the start of aortic angiography, performed to detect extravasation before UAE, to the completion of aortic angiography, confirming hemostasis after UAE.

Initial clinical UAE success was defined as achieving hemostasis with the first UAE procedure. Final clinical UAE success was defined as achieving hemostasis, with or without repeat UAE, without requiring any surgical intervention. The initial and final clinical UAE success rates were the proportions of women with PPH who achieved hemostasis with the initial UAE and with either the initial or repeat UAE, respectively, among those who underwent UAE during both periods.

In the present study, the PPH patients who had a wholly or partially retained placenta, or whose PPH was followed by manual removal of the placenta—a condition referred to as clinical placenta accreta spectrum (PAS)—were classified as having ‘Tissue’-related PPH.

Additionally, patients who received UBT at previous hospitals or clinics were assigned to the UBT-treated group in the UBT period.

### 2.3. Statistical Analysis

The patients’ clinical characteristics and findings were compared between the pre-UBT and UBT periods. Finally, the use rates of UAE, the initial and final clinical UAE success rates and the initial UAE procedural time, along with their clinical characteristics and findings, were compared between women with PPH who underwent UAE during both periods.

The Mann–Whitney U test or Student’s *t*-test was used to analyze continuous variables, whereas the chi-squared or Fisher’s exact test was applied to categorical variables. A *p*-value of <0.05 was considered statistically significant. If significant differences were identified between the two groups in any of the study’s primary comparisons—UAE use rate, initial clinical UAE success rate, final clinical UAE success rate, and initial UAE procedural time—post hoc power calculations were performed.

All statistical analyses were performed using EZR (Saitama Medical Center, Jichi Medical University, Saitama, Japan), a graphical user interface for R (The R Foundation for Statistical Computing, Vienna, Austria). EZR is a modified version of R Commander, specifically adapted to include statistical functions commonly used in biostatistics.

## 3. Results

From January 2009 to December 2023, 41 women in the pre-UBT period (January 2009–December 2014) and 121 women in the UBT period (January 2015–December 2023) were enrolled.

The clinical characteristics of women with PPH in both periods are shown in Table 1. The estimated blood loss upon arrival at our hospital and the total blood loss until hemostasis was achieved were significantly higher in the pre-UBT period than in the UBT period (*p* < 0.01). The lowest plasma fibrinogen levels were significantly lower (*p* < 0.05) and the volumes of RBC and FFP transfusions required until achieving hemostasis were significantly greater in the pre-UBT period (*p* < 0.01).

**Figure 2:**
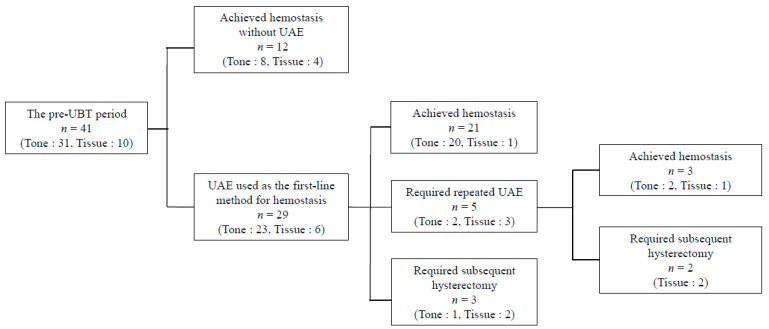
Flowchart of treatment modalities and causes of postpartum hemorrhage during the pre-uterine balloon tamponade period (January 2009–December 2014). Abbreviations: UBT, uterine balloon tamponade; UAE, uterine artery embolization.

The flowchart of the treatment modalities for the pre-UBT period is shown in Figure 2. Among 41 women referred to our hospital for PPH during the pre-UBT period [due to “Tone” (*n* = 31) and “Tissue” (*n* = 10)], 12 (“Tone”, 8; “Tissue”, 4) achieved hemostasis without UAE, while the remaining 29 (“Tone”, 23; “Tissue”, 6) received UAE. Among these 29 women, 21 (“Tone”, 20; “Tissue”, 1) achieved hemostasis after the initial UAE, 3 (“Tone”, 2; “Tissue”, 1) achieved hemostasis after undergoing repeat UAE, and 5 (“Tone”, 1; “Tissue”, 4) required subsequent hysterectomy. During the pre-UBT period, the use rate of UAE was 70.7% (29/41). Patients who underwent UAE during this period were classified as the pre-UBT/UAE group. In this group, the clinical success rates of the initial and final UAE were 72.4% (21/29) and 82.7% (24/29), respectively.

**Figure 3:**
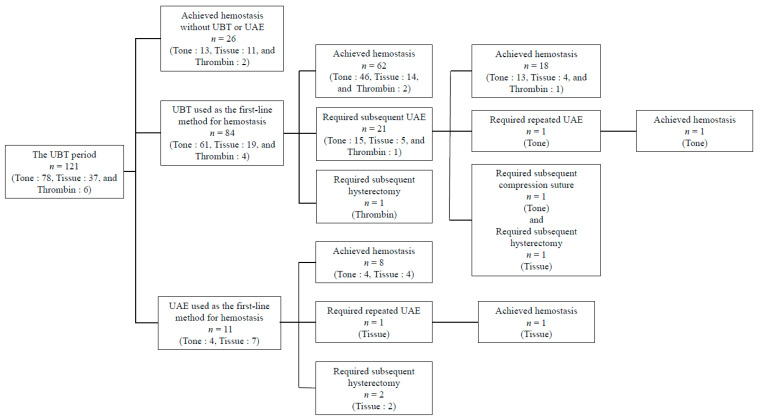
Flowchart of the treatment modalities and causes of postpartum hemorrhage in the uterine balloon tamponade period (January 2015–December 2023). Abbreviations: UBT, uterine balloon tamponade; UAE, uterine artery embolization.

Flowchart of the treatment modalities in the UBT period is shown in Figure 3. Among the 121 women referred to our hospital for PPH during the UBT period (due to “Tone”, 78; “Tissue”, 37; “Thrombin”, 6), 26 (“Tone”, 13; “Tissue”, 11; “Thrombin”, 2) achieved hemostasis without UBT or UAE, 84 (“Tone”, 61; “Tissue”, 19; “Thrombin”, 4) received UBT, and the remaining 11 (“Tone”, 4; “Tissue”, 7) received UAE without undergoing UBT because of persistent massive hemorrhage.

Among the 84 women who received UBT, 60 underwent the procedure at the referring hospitals or clinics, whereas the remaining 24 received UBT after arriving at our institution. In addition, Atom uterine hemostatic balloons were used in 2 women, cervical ripening balloon catheters were used as substitutes for UBT in 3 women, and the remaining 79 women received the Bakri^®^ Postpartum Balloon. The median balloon inflation volume was 150 mL, with a range of 60 to 500 mL. The optimal balloon inflation volume for successful hemostasis could not be determined. Although the receiver operating characteristic analysis suggested that an inflation volume of 180 mL may be optimal for achieving hemostasis, the AUC (0.583; 95% CI, 0.433–0.733) was low, indicating poor reliability.

Among these 84 women who underwent UBT, 21 (“Tone”, 15; “Tissue”, 5; “Thrombin”, 1) required UAE. Among them, 18 (“Tone”, 13; “Tissue”, 4; “Thrombin”, 1) achieved hemostasis after the initial UAE, 1 (“Tone”, 1) achieved hemostasis after undergoing repeat UAE, and 2 required subsequent surgical intervention. Of the 11 women who underwent UAE without attempting UBT, 8 (“Tone”, 4; “Tissue”, 4) achieved hemostasis after the initial UAE, 1 (“Tissue”, 1) achieved hemostasis after undergoing repeat UAE, and 2 (“Tissue”, 2) required subsequent hysterectomy. In the UBT period, the use rate of UAE was 26.4% (32/121). Patients who underwent UAE during this period were classified as the UBT/UAE group. In this group, the initial and final clinical success rates of UAE were 81.3% (26/32) and 87.5% (28/32), respectively.

As a result, the use rate of UAE among patients referred to our hospital for PPH was significantly higher in the pre-UBT period than in the UBT period (70.7% vs. 26.4%; *p* < 0.01; post hoc power, 99.9%). Multivariable logistic regression analysis, adjusted for maternal age, primiparity, pregnancy following ART, gestational weeks at delivery, and cesarean delivery, demonstrated that the introduction of UBT was independently associated with a reduction in the use of UAE (adjusted odds ratio (OR), 0.15 [95% confidence interval (95%CI): 0.05–0.39]; *p* < 0.01). However, no significant difference in the clinical success rates of initial (72.4% vs. 81.3%; *p* = 0.55) and final (82.8% vs. 87.5%; *p* = 0.72) UAE were observed between the pre-UBT/UAE and UBT/UAE groups.

Additionally, in the UBT/UAE group, the initial clinical success rates of UAE among patients who underwent UAE without attempting UBT and those who underwent UAE after UBT were 72.7% (8/11) and 85.7% (18/21), respectively (*p* = 0.39). Moreover, the final clinical success rates were 81.8% (9/11) and 90.5% (19/21), respectively, showing no significant difference (*p* = 0.72).

Table 2 presents the clinical characteristics of the patients with PPH requiring UAE. Twenty-nine and 32 women were assigned to the pre-UBT/UAE and UBT/UAE groups, respectively. The GWs at delivery in the pre-UBT/UAE group were later than in the UBT/UAE group (*p* < 0.05). The estimated blood loss until arrival at our hospital was significantly higher in the pre-UBT/UAE group than in the UBT/UAE group (*p* < 0.05). Moreover, the initial UAE procedural time tended to be longer in the UBT/UAE group than in the pre-UBT/UAE group (*p* = 0.07).

Table 3 summarizes the causes of PPH among patients in both the pre-UBT/UAE and UBT/UAE groups according to the 4Ts, as well as the hemostatic treatment modalities applied. The initial clinical success rates of UAE for “Tone” cases were 87.0% (20/23) and 89.5% (17/19) in the pre-UBT/UAE and UBT/UAE groups, respectively, and the final clinical success rates of UAE were 95.7% (22/23) and 94.7% (18/19), respectively. Contrarily, the initial clinical success rates of UAE for “Tissue” cases were 16.7% (1/6) and 66.7% (8/12) in the pre-UBT/UAE and UBT/UAE groups, respectively, and the final clinical success rates of UAE were 33.3% (2/6) and 75.0% (9/12), respectively.

Table 4 presents the clinical characteristics and courses of women who did not achieve hemostasis with the initial or repeat UAE in both the pre-UBT/UAE and UBT/UAE groups. In the UBT/UAE group, two patients with PPH due to “Tone” achieved hemostasis with UAE but subsequently required hysterectomy during the clinical course: one patient achieved hemostasis with the initial UAE but underwent hysterectomy for uterine necrosis on postpartum day 11, and the other achieved hemostasis with repeat UAE but underwent hysterectomy the following day after being diagnosed with uterine infection.

## 4. Discussion

In the present study, the widespread adoption of UBT was associated with a reduced rate of UAE, even after adjustment for maternal age, primiparity, pregnancy following ART, gestational weeks at delivery, and cesarean delivery (adjusted OR, 0.15 [95% CI, 0.05–0.39]; *p* < 0.01). However, it did not significantly affect the success rate or procedural time of UAE for the management of PPH. Furthermore, there was no difference in the initial clinical success rate of UAE for “Tone”-related PPH cases between the pre-UBT/UAE and UBT/UAE groups (87.0% vs. 89.5%, *p* = 1.0). In “Tissue”-related PPH cases, the initial clinical success rate of UAE was higher in the UBT/UAE group than in the pre-UBT/UAE group (66.7% vs. 16.7%), although this difference was not significant (*p* = 0.13).

Consistent with our findings, several retrospective studies have reported that the introduction of UBT was associated with a reduction in the rate of UAE [18,19,20]. A meta-analysis further demonstrated a significant decrease in UAE following the implementation of UBT (1.9% after UBT introduction vs. 6.3% before; RR, 0.29; 95% CI, 0.14–0.63) [21]. UAE can cause several complications, including neuropathy, organ ischemia or infarction, and even uterine necrosis [8,13,15]. During our study period, one patient required a hysterectomy due to uterine necrosis following UAE. Moreover, UAE for PPH may increase the risk of PAS and PPH in subsequent pregnancies [16]. The implementation of UBT has been associated with a decreased rate of UAE for PPH [19]. In the present study, the introduction of UBT was associated with a reduced use rate of UAE (70.7% vs. 26.4%; *p* < 0.01; post hoc power, 99.9%). We believe that the widespread use of UBT may therefore reduce the number of PPH patients requiring UAE, consequently, UAE-related complications.

The 2017 Japanese guideline on managing critical obstetrical hemorrhagic cases recommends early UBT and prompt transfer to tertiary medical centers for women treated at maternity hospitals or clinics with a shock index of ≥1 [17]. The guideline suggests that early UBT and maternal transfer can reduce blood loss. Indeed, in the present study, the estimated blood loss upon arrival at our hospital was significantly lower during the UBT period than during the pre-UBT period (median, 2000 vs. 2570 g, *p* < 0.01). Conversely, a study enrolling patient with in-hospital-onset PPH after vaginal delivery reported no significant difference in total blood loss before and after the introduction of UBT [22]. UBT may demonstrate its true value as part of the initial management of hemostasis for patients with PPH in primary medical institutions, such as maternity clinics or hospitals, by buying time until arrival at tertiary medical centers.

The UBT success rate in our study was 73.8%, which is lower than the previously reported rates of 83.0–88.9% [21,23,24]. This discrepancy may be partly explained by the following reason. Because our study mainly enrolled patients with PPH for whom hemostasis had been difficult to achieve at previous primary medical institutions, it is likely that our cohort included a larger proportion of patients with severe PPH compared with those in earlier studies.

In addition, we were unable to identify the optimal balloon inflation volume required for successful hemostasis. Consistent with our findings, a previous study reported no significant difference in balloon inflation volume between the UBT success and failure groups [23].

According to the World Health Organization (WHO) recommendation for UBT in the management of PPH [25], UBT is indicated for women with “Tone”-related PPH, specifically uterine atony, that is resistant to standard primary treatments, including uterotonics and tranexamic acid. In the present study, UBT was used in 61 patients with “Tone”-related PPH, with 46 patients (75.4%) achieving hemostasis.

Contrarily, the WHO recommends excluding “Tissue”-related PPH, including those involving retained products of conception (RPOC), from UBT indications because UBT is considered ineffective in these cases [24,25,26]. However, in the present study, 14 (73.7%) of the 19 cases with “Tissue”-related PPH who received UBT achieved hemostasis without additional procedures. Among the 14 cases in which hemostasis was achieved with UBT alone, 13 occurred after the manual removal of the entire placenta and one after the partial manual removal of the placenta; this presentation was referred to as clinical PAS. Conversely, among the five cases in which UBT failed to achieve hemostasis, three involved PPH following manual placenta removal and two involved PPH due to a retained whole placenta. These findings suggest that UBT can be effective for “Tissue”-related PPH, particularly in patients whose PPH is caused by clinical PAS [27]. However, if UBT appears ineffective at achieving hemostasis in patients with “Tissue”-related PPH, UAE or hysterectomy should be performed without delay.

UAE is widely recognized as an effective hemostatic method for PPH that can help preserve the uterus [13]. In our study, 18 (85.7%) out of the 21 patients in whom UBT failed to achieve hemostasis were successfully treated with subsequent UAE. Therefore, UAE should be considered for patients with UBT-resistant PPH.

On the other hand, among women with PPH who underwent UAE, the initial UAE procedural times tended to be longer during the UBT period than during the pre-UBT period (median, 61 vs. 46 min; *p* = 0.07). This tendency may be explained by the higher proportion of PPH cases with difficult-to-achieve hemostasis during the UBT period, as patients with UBT-resistant PPH often require longer procedural times to achieve hemostasis compared with those treated before the implementation of UBT. This study is the first to provide a comparative analysis of initial UAE procedural times in the pre- and post-UBT periods; nevertheless, the small sample size warrants cautious interpretation of the results.

In the present study, the final success rates of UAE were 82.8% and 87.5% in the pre-UBT/UAE and UBT/UAE groups, respectively, which were equivalent to previous reports (82.4–90.5%) [13,28,29]. In our study, the final success rates of UAE for “Tone”-related PPH were 95.7% and 94.7% in the pre-UBT/UAE and UBT/UAE groups, respectively, which were higher than those described in previous reports (86.7–93.1%) [28,29]. Contrarily, the final UAE success rates of UAE for “Tissue”-related UBT were 33.3% and 75.0% in the pre-UBT/UAE and UBT/UAE groups, respectively, which were lower than those described in previous reports (80.0–90.2%) [28,29,30]. Moreover, 7 (77.8%) of the 9 patients in whom the initial or repeat UAE failed to achieve hemostasis had PAS, a major cause of RPOC (1 case of increta and 3 cases of accreta in the pre-UBT period and 2 cases of accreta and 1 clinical PAS in the UBT period). “Tissue”-related PPH, including RPOC, is known to be associated with UAE failure [31]. Although, several reports have proposed UAE as a promising initial therapeutic option for patients with PPH caused by PAS who wish to preserve the uterus [30,32]. As noted above, for “Tissue”-related PPH, particularly in cases with PAS, both UBT and UAE have reduced success rates, and when PAS is suspected it is necessary to proceed without delay to alternative hemostatic measures.

The present study has some limitations. First, the sample size was relatively small. Second, it was a retrospective study. Finally, the treatment approach for each PPH patient was determined individually by the attending obstetricians.

## 5. Conclusions

Our study indicates that the widespread adoption of UBT can reduce the number of women with PPH requiring UAE. Although the introduction of UBT may not change the success rates of UAE, the UAE procedural times may be prolonged. Our study results provide valuable insights for obstetricians and interventional radiologists.

## Figures and Tables

**Table 1:** Clinical characteristics of women with postpartum hemorrhage in the pre-UBT and UBT periods.

Clinical Findings	Pre-UBT Period (*n* = 41)	UBT Period (*n* = 121)	*p*-Value
Age (years)	34 (21–41)	34 (20–42)	0.76
Primiparous	24 (58.5%)	67 (55.4%)	0.46
Pregnancy following ART	16 (39.0%)	47 (38.8%)	1.00
GWs at delivery	39 (30–41)	39 (29–42)	0.75
Cesarean delivery	13 (31.7%)	23 (19.0%)	0.087
Causes of PPH in the “4Ts”			
Tone	31 (75.6%)	78 (64.5%)	0.25
Tissue	10 (24.4%)	37 (30.5%)	0.55
Thrombin	0 (0%)	6 (5.0%)	0.34
Upon arrival at our hospital			
Estimated blood loss (g)	2570 (1100–5040) **	2000 (900–6470) **	<0.01
Hemoglobin concentrations (g/dL)	7.1 ± 1.7	7.2 ± 1.9	0.66
Plasma fibrinogen levels (mg/dL)	184 ± 84	211 ± 92	0.10
Until achieving hemostasis			
Estimated total blood loss (g)	3500 (1400–10,240) **	2600 (900–8950) **	<0.01
Lowest hemoglobin concentrations (g/dL)	6.4 ± 1.3	6.7 ± 1.6	0.21
Lowest plasma fibrinogen levels (mg/dL)	161 ± 71 *	193 ± 84 *	<0.05
Blood transfusion	38 (92.7%)	95 (78.5%)	0.057
Amounts of RBC transfusion (units)	10 (0–36) **	8 (0–34) **	<0.01
Amounts of FFP transfusion (units)	10 (0–34) **	4 (0–34) **	<0.01

Notes: Data are expressed as medians (ranges), averages ± standard deviation, or numbers (percentages). * and ** indicate significant differences between the pre-UBT and UBT groups, corresponding to *p*-values of <0.05 and <0.01, respectively. Abbreviations: UBT, uterine balloon tamponade; ART, assisted reproductive technology; GWs, gestational weeks; PPH, postpartum hemorrhage; RBC, red blood cells; FFP, fresh frozen plasma.

**Table 2:** Clinical characteristics of the patients with postpartum hemorrhage requiring uterine artery embolization in the pre-UBT/UAE and UBT/UAE groups.

Clinical Findings		Pre-UBT/UAE Group (*n* = 29)	UBT/UAE Group (*n* = 32)	*p*-Value
Age (years)		34 ± 4.5	34 ± 5.5	0.85
Primiparous		18 (62.1%)	19 (59.4%)	0.79
Pregnancy following ART		13 (44.8%)	16 (50.0%)	0.79
GWs at delivery		40 (37–41) *	39 (29–42) *	<0.05
Cesarean delivery		8 (27.6%)	9 (28.1%)	0.78
Upon arrival at our hospital				
	Estimated blood loss (g)	2620 (1100–5040) *	2000 (1000–6470) *	<0.05
	Hemoglobin concentrations (g/dL)	7.1 ± 1.5	6.9 ± 1.8	0.71
	Plasma fibrinogen levels (mg/dL)	172 ± 83	189 ± 100	0.76
Until achieving hemostasis				
	Initial UAE procedural time (minutes)	46 (9–178)	61 (24–139)	0.07
	Estimated total blood loss (g)	4130 (1660–10,240)	3530 (1500–8950)	0.38
	Lowest hemoglobin concentrations (g/dL)	6.2 ± 1.2	6.3 ± 1.3	0.76
	Lowest plasma fibrinogen levels (mg/dL)	147 (34–364)	150 (29–351)	0.48
	Blood transfusion	29 (100.0%)	31 (96.9%)	1.00
	Amounts of RBC transfusion (units)	13 (4–36)	13 (0–34)	0.99
	Amounts of FFP transfusion (units)	12 (4–34)	9 (0–34)	0.17

Notes: Data are expressed as medians (ranges), averages ± standard deviation, or numbers (percentages). * indicates significant differences between the pre-UBT/UAE and UBT/UAE groups, corresponding to *p*-values of <0.05. Abbreviations: UBT, uterine balloon tamponade; ART, assisted reproductive technology; GWs, gestational weeks; UAE, uterine artery embolization; RBC, red blood cells; FFP, fresh frozen plasma.

**Table 3:** Causes of postpartum hemorrhage and final treatment modalities used to achieve hemostasis in the pre-UBT/UAE and UBT/UAE groups.

			Causes of PPH in the “4Ts”		
			Tone	Tissue	Thrombin
In the pre-UBT/UAE group (*n* = 29)					
	Achieved hemostasis with the initial UAE		20 (87.0%)	1 (16.7%)	0 (ND)
	Achieved hemostasis with repeated UAE		2	1	0
	Achieved hemostasis with hysterectomy		1	4	0
	Total		23	6	0
The UBT/UAE group (*n* = 32)					
	Achieved hemostasis with the initial UAE		17 (89.5%)	8 (66.7%)	1 (100%)
		UAE following UBT	13	4	1
		UAE without preceding UBT	4	4	0
	Achieved hemostasis with repeated UAE		1	1	0
		UAE following UBT	1	0	0
		UAE without preceding UBT	0	1	0
	Achieved hemostasis with compression suture		1	0	0
	Achieved hemostasis with hysterectomy		0	3	0
	Total		19	12	1

Abbreviations: UAE, uterine artery embolization; ND, not determined; UBT, uterine balloon tamponade.

**Table 4:** Clinical characteristics and outcomes of women who did not achieve hemostasis with the initial or repeat uterine artery embolization in the pre-UBT/UAE and UBT/UAE groups.

No.	Age (y)	Gravidity (No)/Parity (No.)	Pregnancy Following ART	GWs at Delivery/ Delivered Mode	Upon Arrival at Our Hospital						Until Achieving Hemostasis							Final Diagnosis of the Causes of PPH
					Clinical Findings	Estimated Blood Loss (g)	Hb Level (g/dL)	Plasma Fibrinogen Level (mg/dL)	UBT		Estimated Total Blood Loss (g)	The Initial UAE Procedure Time (min)	Lowest Hb Level (g/dL)	Lowest Plasma Fibrinogen Level (mg/dL)	Amount of RBC/FFP Transfusion (Units)	UBT	Final Procedure to Achieve Hemostasis	
In the pre-UBT/UAE group																		
1	38	1/1	No	40/VD	After manual removal of placenta	2500	6.5	157	N/A		3500	29	4.9	140	10/12	N/A	Hysterectomy	Increta
2	37	2/2	Yes	40/VD	After manual removal of placenta	3400	5.4	188	N/A		5900	78	4.5	158	14/14	N/A	Hysterectomy	Accreta
3	25		Yes	40/VD	Retained whole placenta	2660	9.5	148	N/A		5500	24	5.5	58	36/20	N/A	Hysterectomy	Accreta
4	36	1/1	Yes	37/CS	After manual removal of placenta	3000	5.4	95	N/A		7000	28	5.4	95	20/20	N/A	Hysterectomy	Accreta
5	37	2/2	No	40/VD	Uterine atony	4000	5.8	75	N/A		7000	104	5.8	75	30/34	N/A	Hysterectomy	Atonic bleeding
In the UBT/UAE group																		
6	37	2/2	Yes	37/VD	Retained whole placenta	2730	8.5	126	No		8500	65	7.8	126	24/28	No	Hysterectomy	Accreta
7	35	2/2	Yes	37/VD	Retained whole placenta	2400	6.1	301	No		5960	78	5.6	149	14/10	No	Hysterectomy	Accreta
8	28	1/1	No	37/CS	After manual removal of placenta	6470	7.0	84	Yes		6840	55	7.0	84	20/24	Yes	Hysterectomy	Clinical PAS
9	36	1/1	Yes	38/CS	Uterine atony	1790	7.4	263	Yes		5500	24	6.9	124	24/28	Yes	Compression suture	Atonic bleeding

Abbreviations: PPH, postpartum hemorrhage; UAE, uterine artery embolization; ART, assisted reproductive technology; GW, gestational week; Hb, hemoglobin; UBT, uterine balloon tamponade; RBC, red blood cells; FFP, fresh frozen plasma; VD, vaginal delivery; CS, cesarian delivery; PAS, placenta accreta spectrum; N/A, not applicable.

## Data Availability

The raw data supporting the conclusions of this article is not publicly available due to patient privacy and ethical restrictions. However, they can be obtained from the corresponding author upon reasonable request.

## Abbreviations

The following abbreviations are used in this manuscript:

PPH	postpartum hemorrhage
FFP	fresh frozen plasma
UAE	uterine artery embolization
WHO	World Health Organization
UBT	uterine balloon tamponade
ART	assisted reproductive technology
GWs	gestational weeks
RBC	red blood cells
RPOC	retained products of conception
PAS	placenta accreta spectrum
